# Mechanistic Insights into the Mechanism of Inhibitor Selectivity toward the Dark Kinase STK17B against Its High Homology STK17A

**DOI:** 10.3390/molecules27144655

**Published:** 2022-07-21

**Authors:** Chang Liu, Yichi Zhang, Yuqing Zhang, Zonghan Liu, Feifei Mao, Zongtao Chai

**Affiliations:** 1 Department of Hepatic Surgery VI, Eastern Hepatobiliary Surgery Hospital, Second Military Medical University, Shanghai 200433, China; viclchang@163.com (C.L.); liuzonghan@aliyun.com (Z.L.); 2 Department of Transplantation, Xinhua Hospital Affiliated to Shanghai Jiao Tong University, School of Medicine, Shanghai 200092, China; zhangyichi@xinhuamed.com.cn; 3 MD Cancer Center, Yue Yang Hospital of Integrative Traditional Chinese and Western Medicine, Affiliated to Shanghai University of Traditional Chinese Medicine, Shanghai 200437, China; perizhangyuqing@163.com; 4 Tongji University Cancer Center, Shanghai Tenth People’s Hospital, School of Medicine, Tongji University, Shanghai 200072, China; 5 Department of Hepatic Surgery, Shanghai Geriatric Center, Shanghai 201104, China; 6 Liver Cancer Institute, Zhongshan Hospital, Fudan University, Key Laboratory of Carcinogenesis and Cancer Invasion (Fudan University), Ministry of Education, Shanghai 200032, China

**Keywords:** STK17B, STK17A, molecular dynamics simulation, binding free energy, binding selectivity

## Abstract

As a member of the death-associated protein kinase (DAPK) family, STK17B plays an important role in the regulation of cellular apoptosis and has been considered as a promising drug target for hepatocellular carcinoma. However, the highly conserved ATP-binding site of protein kinases represents a challenge to design selective inhibitors for a specific DAPK isoform. In this study, molecular docking, multiple large-scale molecular dynamics (MD) simulations, and binding free energy calculations were performed to decipher the molecular mechanism of the binding selectivity of PKIS43 toward STK17B against its high homology STK17A. MD simulations revealed that STK17A underwent a significant conformational arrangement of the activation loop compared to STK17B. The binding free energy predictions suggested that the driving force to control the binding selectivity of PKIS43 was derived from the difference in the protein–ligand electrostatic interactions. Furthermore, the per-residue free energy decomposition unveiled that the energy contribution from Arg41 at the phosphate-binding loop of STK17B was the determinant factor responsible for the binding specificity of PKIS43. This study may provide useful information for the rational design of novel and potent selective inhibitors toward STK17B.

## 1. Introduction

Protein kinases catalyze the transfer of the γ-phosphate moiety of ATP to a variety of different substrates such as proteins and lipids. This biological process, also called phosphorylation, plays an essential role in the regulation of versatile cellular functions, including signal transduction, metabolism, apoptosis, cell proliferation, and inflammation [1]. Abnormal protein kinase activity is, therefore, associated with the pathogenesis of numerous human diseases, including cancer, diabetes, inflammation, Parkinson’s disease, and Alzheimer’s disease [2]. Thus, the protein kinase family has become one of the most important classes of drug targets in the pharmaceutical industry. Accordingly, until now, a total of 72 small-molecule kinase inhibitors have been approved by the US Food and Drug Administration (FDA) [3,4,5].

The human protein kinase gene family contains 518 members. Despite the availability of many FDA-approved protein kinase inhibitors, these approved drugs approximately target 45 kinase members such as epidermal growth factor receptor (EGFR), breakpoint cluster region (BCR)-abelson tyrosine kinase (ABL), mesenchymal phosphoinositide 3kinase (PI3K), cyclin-dependent kinase (CDK), epithelial transition factor receptor (MET), and anaplastic lymphoma kinase (ALK), representing only ~30% of the human kinome and suggesting that a majority of this drug class remains unexplored [6]. A key challenge in the development of kinase inhibitors is largely derived from the poor kinase selectivity of inhibitors, because of the highly conserved ATP-binding site among distinct kinase members, specially the homologous kinase subfamily [7]. Thus, understanding the molecular underpinnings of kinase inhibitor selectivity can contribute to the development of drugs aiming for the unexplored targets.

STK17B represents a classical example for selective inhibitor development, belonging to the death-associated protein kinase (DAPK) family, one of the largest families of serine/threonine kinases [8]. It has a highly homologous member, STK17A. STK17B plays an important role in the regulation of cellular apoptosis [9]. Abnormality of this function is involved in most cancers, owing to its attenuated expression or gene silencing. It has been well-established that overexpression of STK17B has closely associated with the tumorigenesis of hepatocellular carcinoma (HCC) and breast cancer [10], and inhibition of STK17B inhibited HCC tumorigenesis and metastasis via AKT/GSK3β/Snail signaling [11]. The importance of STK17B in cellular functions renders it as a promising therapeutic target in drug development [12]. 

**Figure 1:**
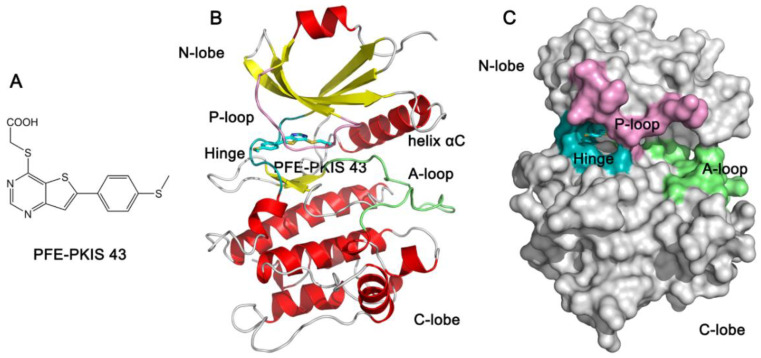
(**A**) Chemical structure of PFE-PKIS-43. Cartoon (**B**) and surface (**C**) representations of STK17B−PFE-PKIS-43 structural complex (PDB ID: 6Y6F). The phosphate-binding loop (P-loop), the hinge, and the activation loop (A-loop) are colored by pink, cyan, and lime, respectively.

Despite the significance of STK17B in oncology, the selective inhibitors of STK17B, as chemical probes to disrupt its catalytic function in cells, are currently unavailable. In fact, the previously reported inhibitors such as quercetin, dovitinib, and benzofuranone are non-selective or modestly selective regarding the inhibition of STK17B [13,14,15,16,17,18]. Recently, Picado et al. have discovered a cell that is an active STK17B inhibitor, thieno[3,2-d] pyrimidine PFE-PKIS 43 (named PKIS43 hereafter) (Figure 1A). KinaseSeeker split luciferase binding assays showed that compared to the control (DMSO) at 1 μM, PKIS43 exhibited 90% inhibition at STK17B with an IC_50_ of 20 ± 3.9 nM, while it exhibited 10% inhibition at the homologous protein kinase STK17A [19]. Thus, PKIS43 showed remarkable potency and high selectivity toward STK17B against STK17A. PKIS43 binds to the ATP-binding site of STK17B and interacts with both the N- and C-lobes of the kinase (Figure 1B,C). In a recent study, we have used multi-microsecond-length molecular-dynamics (MD) simulations of STK17B in three different states (ligand-free, ADP-bound, and ligand-bound states) to uncover the conformational plasticity of the phosphate-binding loop (P-loop) [20]. However, how the P-loop of STK17B and STK17A affects inhibitor selectivity is still unexplored. Moreover, the STK17A−PKIS43 structural complex is still unavailable. Given the highly conserved ATP-binding site between STK17B and STK17A, how the molecular mechanism of the selective inhibition of STK17B against STK17A by PKIS43 functions remains unclear.

Here, we performed molecular docking and MD simulations to decipher the selective inhibition mechanism of a specific STK17B isoform. Molecular docking of PKIS43 to the ATP-binding site was performed, and then four independent runs of microsecond MD simulations of STK17B–PKIS43 and STK17A–PKIS43 complexes were performed in an explicit water environment. Coupled with post-trajectory analysis such as principal component analysis, dynamical cross-correlation analysis, community network analysis, and binding free energy calculations, we selectivity revealed the critical residues responsible for the control of the inhibitor. We expect that the comprehensive analysis of binding interactions between PKIS43 and STK17B and STK17A, from both the structural and energetic perspectives, can provide a useful static in the rational design of novel selective STK17B inhibitors.

## 2. Results and Discussion

### 2.1. Molecular Docking Study

**Figure 2:**
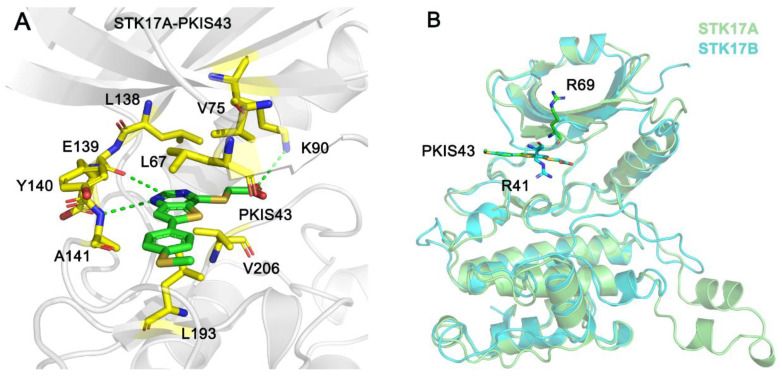
(**A**) The predicted interactions between STK17A and PKIS43. Hydrogen bonds or salt bridge are shown by green dotted lines. (**B**) Structural superimposition between STK17A−PKIS43 and STK17B−PKIS43 complexes.

To date, the X-ray crystal structure of STK17B in complex with PKIS43 has been determined, but the STK17A−PKIS43 structural complex is still unavailable. Thus, the molecular docking method was used to construct the STK17A−PKIS43 complex based on the known structural complex of STK17A−CKJB68. The molecular docking method has been widely applied to model protein–ligand or protein–protein interactions. In our study, CKJB68 was removed from the STK17A structure, and PKIS43 was then docked into the ATP-binding site of halo STK17A using AutoDock 4.2 software [21]. A total of 100 independent runs were performed, thereby generating 100 different docking conformations for PKIS43 in the ATP-binding site of STK17A. Cluster conformational analysis was performed to classify the 100 docked conformations of PKIS43 into subgroups based on 1.0 Å root-mean-square deviation (RMSD) criterion. This result showed that PKIS43 mainly adopted one preferred orientation (88%) in the ATP-binding site of STK17A. Furthermore, in the largest cluster, we selected the docked complex with the lowest binding free energy of PKIS43 to STK17A to describe the predicted interactions between STK17A and PKIS43. As shown in Figure 2A, the pyrimidine moiety of PKI43 forms a hydrogen bond with the backbone amide of Ala141 and a weak hydrogen bond (C-H---O) with the backbone carbonyl oxygen of Glu139 in the hinge region. The carboxylate moiety of PKI43 is involved in a salt bridge with the catalytic Lys90 in the strand β3; the latter forms a critical salt bridge with Glu108 in the helix αC from the N-lobe. This key salt-bridge interaction is conserved in the kinome and is responsible for maintaining the catalytic activity of protein kinases. In addition, PKI43 engages in extensive hydrophobic interactions with Leu67, Val75, Leu138, Tyr140, Leu193, and Val206. We further superimposed the two complexes of STK17B−PKIS43 and STK17A−PKIS43 and found that the conformational arrangement of PKI43 in the ATP-binding site of both complexes was similar (Figure 2B), indicating the feasibility and accuracy of the molecular docking technique to reproduce the protein–ligand interactions. Notably, the conformation of Arg41 in STK17B protrudes into the ATP-binding site and forms salt bridges with the carboxylic acid of PKIS43. In contrast, the corresponding residue is Arg69 in STK17A, where it positions itself opposite to the ATP-binding site and has no interaction with PKIS43.

### 2.2. Overview of Simulated Systems

**Figure 3:**
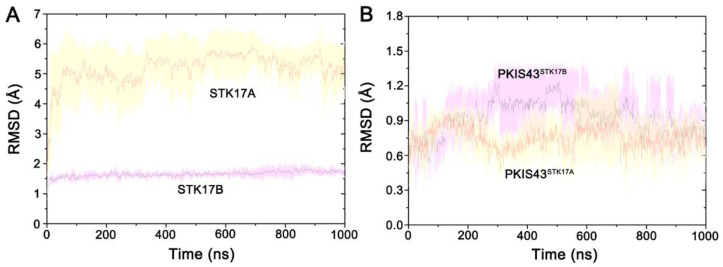
(**A**) The Cα atoms of root-mean-square deviation (RMSD) for STK17B and STK17A averaged over four independent runs. (**B**) The heavy atoms of RMSD for PKIS43 in the ATP-binding site of STK17B and STK17A averaged over four independent runs. Transparent shade represents standard deviations.

MD simulations were performed to probe the conformational dynamics of protein–ligand interactions [22,23,24,25,26,27,28]. In total, four replicas of each 1 μs MD simulations were conducted for both the SKT17B−PKIS43 and SKT17A−PKIS43 complexes, in an explicit water environment. To show the conformational dynamics of both systems during MD simulations, the Cα atoms of RMSD for STK17B and STK17A throughout all four replicas were calculated relative to the initial simulated structures. As shown in Figure 3A, the RMSD plots showed that STK17B reached equilibrium at the beginning of the simulations, with an RMSD value of 1.66 ± 0.14 Å. However, STK17A reached equilibrium after ~100 ns simulations and had a larger RMSD value (5.19 ± 0.91 Å) than that of STK17B, implying that compared to STK17B, STK17A underwent a significant conformational arrangement during MD simulations. The structural overlap of STK17A between the starting and the equilibrated structures revealed that the markedly conformational arrangements of STK17A were largely derived from the flexibilities of the A-loop and the C-terminal end. To further show the stability of PKIS43 in the ATP-binding site of both kinases, we monitored the heavy atoms of RMSD for PKIS43 throughout all four independent runs. As shown in Figure 3B, PKIS43 in both kinases showed subtle deviations, with the small RMSD values of 0.94 ± 0.25 Å and 0.75 ± 0.20 Å in STK17B and STK17A, respectively. This result indicated that PKIS43 was stable in the ATP-binding site of both kinases, which laid the foundation for the subsequent binding free energy calculations.

**Figure 4:**
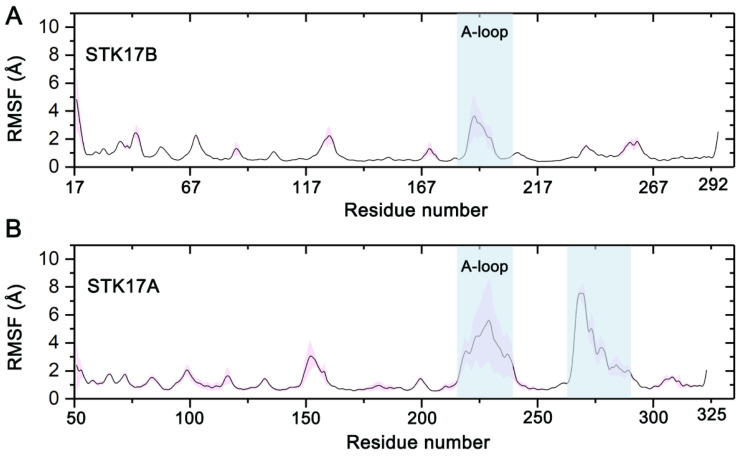
The Cα atoms of root-mean-square fluctuation (RMSF) for STK17B (**A**) and STK17A (**B**) averaged over four independent runs. Transparent shade represents standard deviations.

To further reveal the local residue fluctuations, we calculated the root-mean-square fluctuation (RMSF) of the Cα atoms of each residue in both kinases, using all simulated snapshots from the four replicas. For STK17A, the first 100 ns trajectories were not calculated due to those non-equilibrium snapshots. As shown in Figure 4, the overall fluctuations were conserved in both kinases except the A-loop (residues Asn216−Ser241 in STK17A) and the C-lobe (residues Ile260−Leu290 in STK17A). The RMSD value for the Cα atoms of residues Ile260−Leu290 in STK17A was 2.66 ± 0.35 Å. Compared to STK17B, the two regions showed markedly enhanced conformational fluctuations in the SKT17A. Despite the large conformational dynamics of the A-loop and the C-lobe in STK17A, they had no noticeable effect on the conformational stability of PKIS43 in the ATP-binding site, because the two regions have no direct contact with PKIS43.

### 2.3. Dynamical Cross-Correlation Analysis

**Figure 5:**
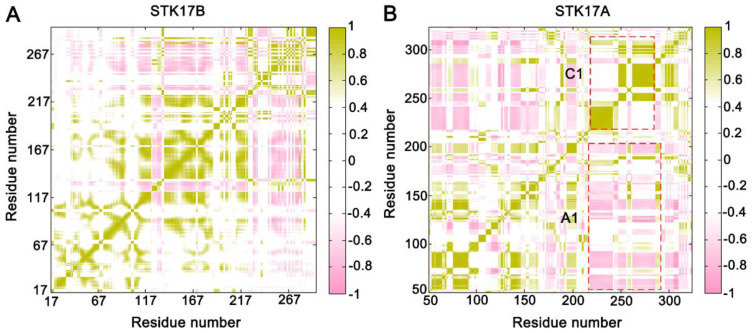
The dynamical cross-correlation matrix (DCCM) of the STK17B (**A**) and STK17A (**B**) systems. Correlated motions with absolute values < 0.4 are ignored for clarity and shown in white. A1 shows anti-correlated motions between the A-loop and the N-terminus, and C1 represents the correlated motions between the A-loop and the C-terminus.

To unveil the dynamic variation of STK17B and STK17A, the inter-residue correlations were analyzed using the dynamical cross-correlation matrix (DCCM) calculations [20,29,30]. Correlation coefficients (*CC_ij_*) between every two Cα atoms (*i* and *j*) were calculated using all snapshots from four replicas, which showed the relationship among the spatially different domains in both the WT and Q597A mutant systems. In the DCCM calculation, the positive *CC_ij_* values (*CC_ij_* > 0) represent the movement of the two residues in the correlated motions, while the negative *CC_ij_* values (*CC_ij_* < 0) mean the movement of the two residues in the anti-correlated motions [31]. As shown in Figure 5, the overall dynamic patterns of STK17B and STK17A were similar. However, the anti-correlated motions (A1) between the A-loop and the N-lobe and the correlated motions (C1) between the A-loop and the C-lobe were strengthened in STK17A compared to STK17B. These results were in agreement with the RMSF analysis, wherein the A-loop and the C-lobe of STK17A showed large conformational fluctuations.

### 2.4. Binding Free Energy Calculations

To reveal the binding selectivity of PKIS43 to STK17B against STK17A, the binding free energies for the two systems were calculated using the MM−GBSA method, based on the 200 snapshots evenly extracted from the last 200 ns MD trajectories. As listed in Table 1, the predicted binding free energies (∆G_binding_) for STK17B−PKIS43 and STK17A−PKIS43 were -33.14 ± 4.04 and -26.13 ± 5.24 kcal/mol, respectively. According to the prediction results, PKIS43 increased the binding affinity by 7.01 kcal/mol to STK17B compared to STK17A. Thus, PKIS43 formed much stronger contacts with STK17B than with STK17A, which suggested that PKIS43 is a selective inhibitor of STK17B and is in agreement with the experimental results. PKIS43 showed a K_d_ = 3.8 nM for STK17B but a K_d_ = 220 nM for STK17A [19]. It should be noted that the difference in the binding affinities of PKIS43 to STK17B and STK17A was largely derived from the difference in the electrostatic interactions (∆E_ele_), as shown in Table 1, while the van der Waals interactions (∆E_vdW_) in both systems were identical.

### 2.5. Key Residues for Binding Selectivity Revealed by Free Energy Decomposition

To unravel the critical residues to determine the binding selectivity of PKIS43, we further calculated the per-residue binding free energies between PKIS43 and STK17B/STK17A using the MM−GBSA free energy decomposition approach. For both systems, the total binding free energy was decomposed, and the residues with energy contributions > −1.0 kcal/mol were selected. As shown in Table 2, the main residue contributions for STK17B/STK17A originated from Leu39/Leu67, Val47/Val75, Ala60/Ala88, Lys62/Lys90, Leu110/Leu138, Tyr112/Tyr140, Ala113/Ala141, Gly116/Gly144, Leu165/Leu193, and Val178/Val206. These 10 residues are quite conserved in the ATP-binding sites of both STK17B and STK17A and have similar energy contributions to the binding of PKIS43. Remarkably, residue Arg41 at the P-loop had a large binding affinity (−5.27 ± 1.30 kcal/mol) to STK17B, while the corresponding residue Arg69 had no binding contribution to STK17A. This is because, in STK17A, the conformation of Arg69 is positioned opposite to the ATP-binding site and forms no interaction with the inhibitor PKIS43. Owing to the more favorable energetic contributions provided by Arg41, it, thus, played a critical role in the determination of binding specificity of PKIS43 toward STK17B over STK17A.

### 2.6. Comparative Binding Modes

**Figure 6:**
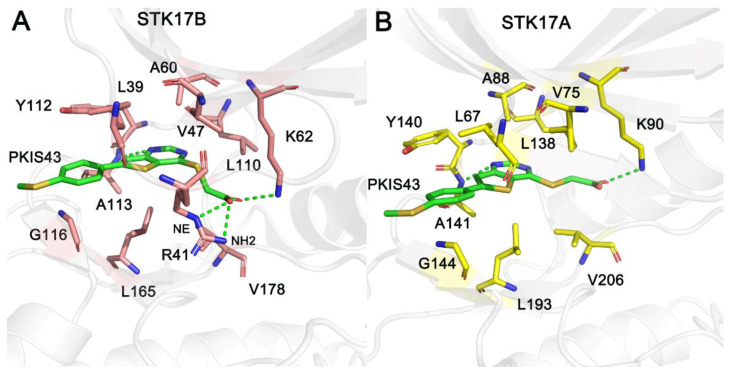
The detailed interaction modes of STK17B–PKIS43 (**A**) and STK17A–PKIS43 (**B**) complexes. The atoms named “NE” and “NH2” of Arg41 in STK17B are labeled. Hydrogen bonds or salt bridges are shown by green dotted lines.

**Figure 7:**
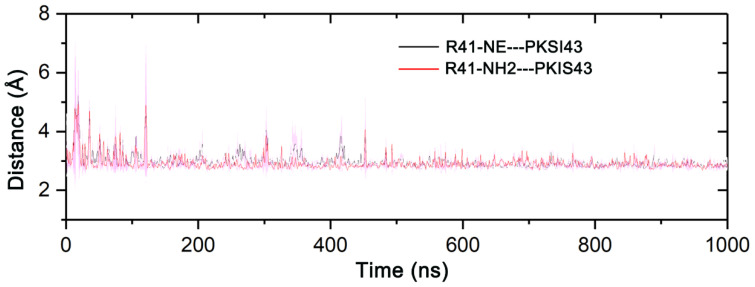
The distances between the NE atom of Arg41 and the carboxylate oxygen atom of PKIS43 as well as between the NH2 atom of Arg41 and the carboxylate oxygen atom of PKIS43, averaged over four independent runs. Transparent shade represents standard deviations.

In order to reveal the detailed binding modes of PKIS43 at the ATP-binding site of STK17B and STK17A as well as to further explain the structure-based origin of the binding selectivity of PKIS43 toward STK17B, we performed the cluster analysis method to extract the most representative structural complexes from the four simulated replicas for both systems [32]. As shown in Figure 6, the backbone amide of Ala113/Ala141 of STK17B/STK17A at the hinge region forms a hydrogen bond with the pyrimidine moiety of PKI43. The catalytic Lys62/Lys90 of STK17B/STK17A is involved in a salt bridge with the carboxylate moiety of PKIS43. In addition to these polar interactions, PKIS43 engages in extensive hydrophobic interactions with the Leu39/Leu67, Val47/Val75, Ala60/Ala88, Leu110/Leu138, Tyr112/Tyr140, Ala113/Ala141, Gly116/Gly144, Leu165/Leu193, and Val178/Val206 of STK17B/STK17A. Overall, these above polar and hydrophobic interactions are conserved in both STK17B and STK17A systems. However, in STK17B, residue Arg41 at the P-loop forms two salt bridges with the carboxylate moiety of PKIS43, which can significantly enhance the binding affinity. In contrast, these salt-bridge interactions are absent in STK17A. To further reveal the stability of the salt-bridge interactions between the Arg41 of STK17B and the carboxylate moiety of PKIS43, we monitored the distances between them using simulated snapshots from the four replicas. As shown in Figure 7, both the distances between the NE atom of Arg41 and the carboxylate oxygen atom of PKIS43 as well as between the NH2 atom of Arg41 and the carboxylate oxygen atom of PKIS43 were stable after ~100 ns simulations, with values of 2.95 ± 0.17 Å and 2.90 ± 0.17 Å, respectively. This result suggested the formation of two salt bridges between the Arg41 of STK17B and PKIS43. Taken together, this can be explained as the origin of binding selectivity of PKIS43 toward STK17B against STK17A, because Arg41 plays an essential role in binding affinity, as shown by per-residue free energy decomposition analysis.

## 3. Materials and Methods

### 3.1. System Preparation

The co-crystal structure of STK17B in complex with PKIS43 was downloaded from the RCSB Brookhaven Protein Data Bank (PDB), with the code 6Y6F [19]. The missing residues Cys187–Ile195 at the activation loop (A-loop) of STK17B were constructed using MODELLER software [33]. The co-crystal structure of STK17A in complex with CKJB68 was retrieved from the PDB, with the code 7QUE. CKJB68 was removed from STK17A, and the remaining protein was named as the halo STK17A. The missing residues Asp148–Glu156 in the halo STK17A were modeled. The partial charges for the inhibitor PFE-PKIS 43 were calculated using the RESP HF/6-31G* method [34] via the antechamber module in AMBER 18 and Gaussian 09 software (Wallingford, UK).

### 3.2. Molecular Docking

Molecular docking of the inhibitor PKIS43 to the ATP-binding site of the halo STK17A (PDB ID: 7QUE) was performed using AutoDock 4.2 software [21]. Polar hydrogen atoms were added to STK17A using AutoDock Tools (ADT). Kollman united atom partial charges were used for PKIS43, and AutoDock atom types for the inhibitor were added through ADT. For the inhibitor, all hydrogen atoms were added and the default root, rotatable bonds, and torsion were used. The 60, 60, and 60 grid points in the x, y, and z directions were set using the spacing value of 0.375 Å through AutoGrid. The grid center was defined at the centroids (−14.14, 2.65, and 30.48 for x, y, and z directions, respectively) of the inhibitor CKJB68 in STK17A. In the docking parameters, a maximum number of 2,500,000 energy evaluations, a maximum number of 27,000 generations, an initial population of 150 randomly placed individuals, a mutation rate of 0.02, and a crossover rate of 0.80 were used. In the production run, 100 independent docking runs were conducted using the Lamarckian genetic algorithm.

### 3.3. MD Simulations

MD simulations of two systems, STK17B–PKIS43 and STK17A–PKIS43 complexes, were performed using AMBER 18 software [35]. The Amber ff14SB force field [36] was used for the protein, and the general amber force field (GAFF) [37] was used for the ligand. A truncated octahedral TIP3P [38] water box with 10 Å was employed to solvate the protein–ligand complex. Two rounds of minimizations were performed, including the steepest descent and conjugate gradient algorithms [39,40,41]. Then, both systems were heated up from 0 to 300 K within 1 ns of MD simulations under the canonical ensemble, using the position restraints of 10 kcal/(mol∙Å^2^) on the solute atoms. Finally, 4 replicas of each 1 μs simulation without any position restraints were performed with random velocities under isothermal isobaric conditions. An integration time step of 2 fs was used. The SHAKE algorithm was used to constrain all bond lengths involving hydrogen atoms [42]. The particle mesh Ewald method was used to treat the long-range electrostatic interactions [43], while a 10 Å non-bonded cut-off was used for the short-range electrostatics and van der Waals interactions.

### 3.4. Dynamical Cross-Correlation Analysis

The dynamical cross-correlation matrix (*CC_ij_*) was calculated to describe the coupling of the motions between the protein residues. *CC_ij_* was calculated using the following equation [44,45,46,47]:

$$C(i,j)=\frac{c(i,j)}{c{\left(i,i\right)}^{1/2}c{\left(j,j\right)}^{1/2}}$$

where *i* and *j* represent the *i*th and *j*th Cα atoms of two residues.

### 3.5. Binding Free Energy Calculations

The molecular mechanisms generalized Born surface area (MM–GBSA) binding energy calculations were performed using the following equations [48,49,50,51,52]:

∆G_binding_ = ∆G_complex_ − [∆G_protein_ + ∆G_ligand_]

∆G_binding_ = ∆E_gas_ + ∆G_solvation_ − T∆S

∆E_gas_ = ∆E_vdW_ + ∆E_ele_

∆G_solvation_ = ∆G_GB_ + ∆G_nonpolar_

∆G_nonpolar_ = γ × SASA + b

The ∆E_vdW_, ∆E_ele_, ∆E_gas_, ∆G_solvation_, ∆G_GB_, and ∆G_nonpolar_ terms were the van der Waals energy, electrostatic energy, gas energy, solvation free energy, polar energy, and nonpolar energy, respectively. The entropy term (T∆S) was not calculated owing to the extremely long durations of normal-mode analysis of large systems [49]. The ΔG_nonpolar_ was calculated using the function of the solvent accessible surface area with a γ value of 0.0072 kcal/(mol∙Å^2^) and a b value of 0 kcal/mol. Per-residue free energy decomposition of the total ∆G_binding_ was then carried out using the same MM–GBSA setting [53,54].

## 4. Conclusions

In the present study, multiple μs MS simulations, DCCM, PCA, and MM−GBSA binding free energy calculations were employed to decipher the molecular basis of the binding selectivity of PKIS43 toward STK17B against its high homology STK17A. The binding selectivity of PKIS43 can be generally assessed by the binding free energies of the means of the MM−GBSA calculations. The binding free energy calculations indicated that the electrostatic interactions controlled the binding selectivity of PKIS43. The energy decomposition analysis suggested that Arg41 at the P-loop played a key role in the binding selectivity of PKIS43 to STK17B, as revealed by the salt-bridge interactions. This predicted residue requires further experimental testing. We anticipate that this study can deepen our understanding of the selective mechanism of STK17B inhibitors and offer beneficial information for the design of novel and potent selective STK17B inhibitors.

## Figures and Tables

**Table 1:** Binding free energy (kcal/mol) between PKIS43 and STK17B and STK17A.

	STK17B–PKIS43	STK17A–PKIS43
∆E_vdW_	−42.31 ± 4.23	−41.67 ± 5.31
∆E_ele_	−16.69 ± 3.60	−11.25 ± 3.63
∆G_SA_	−5.01 ± 0.16	−6.39 ± 0.34
∆G_GB_	30.87 ± 4.72	33.18 ± 5.09
∆G_binding_	−33.14 ± 4.04	−26.13 ± 5.24

**Table 2:** Energy contributions (kcal/mol) of key residues to the binding free energy.

Residues	STK17B	Residues	STK17A
Leu39	−1.94 ± 0.43	Leu67	−2.07 ± 0.41
Arg41	−5.27 ± 1.30	Arg69	0.06 ± 0.01
Val47	−1.06 ± 0.14	Val75	−1.54 ± 0.43
Ala60	−1.01 ± 0.23	Ala88	−0.96 ± 0.28
Lys62	−8.00 ± 0.92	Lys90	−7.71 ± 1.41
Leu110	−1.62 ± 0.31	Leu138	−1.59 ± 0.35
Tyr112	−2.79 ± 0.36	Tyr140	−2.50 ± 0.37
Ala113	−1.65 ± 0.48	Ala141	−1.69 ± 0.40
Gly116	−1.46 ± 0.34	Gly144	−1.54 ± 0.38
Leu165	−2.19 ± 0.23	Leu193	−2.23 ± 0.34
Val178	−2.77 ± 0.46	Val206	−3.12 ± 0.62

## Data Availability

Not applicable.
